# Powers of Insurance Committees

**Published:** 1912-11-09

**Authors:** F. W. Francis

**Affiliations:** Leamington Spa.


### The Editor's Letter-Box.

### Powers of Insurance Committees.

*To the Editor of* The Hospital.

Sir,—Your powerful article of last week on "Insured Persons and Voluntary Hospitals" commands attention; but I find a serious flaw in its argument. At the present time, as vice-chairman of an Insurance Committee, I am painfully aware that we cannot spend one penny without sending to the Commissioners an estimate showing the way we propose to spend it. Now, the Commissioners insist that every penny we spend shall be spent in accordance with the National Insurance Act, and that Act gives no power to Insurance Committees (or the Commissioners either, for that matter) to pay one penny for hospital treatment (except for sanatorium hospital benefit for tuberculosis). The insured person receives the same medical benefit as the old Friendly Society member, and should be treated in the same way—*i.e.*, not be admitted as an in-patient if he can be treated equally well by his own doctor. Public Health Authorities have power to establish hospitals, and in my opinion they are the people to whom deputations should be sent if the medical profession decide (and it is in their power to do so) to refuse free services to any person who is employed (why give free service to their dependants?) except in sudden emergency. Such refusal will force the majority of hospitals in large towns to close their doors, as many thousand pounds of annual subscriptions from working men and sympathisers will immediately cease, and whatever compromise is come to will never be renewed. As an active member of the committee of our hospital here for many years I trust other counsels will prevail.—I am, Sir, your obedient servant,

F. W. Francis.

Leamington Spa.

